# Chitosan/Sodium Alginate Hydrogel for the Release of Berberine as an Algae Suppressant: RSM Optimization and Analysis of Sustained Release Characteristics

**DOI:** 10.3390/gels10090591

**Published:** 2024-09-13

**Authors:** Yingjun Wang, Mengting Wu, Panyang Tang, Dongmei Jiang

**Affiliations:** College of Environment, Sichuan Agricultural University, Chengdu 611130, China; w307043@163.com (M.W.); a1206369655@163.com (P.T.); a863076@163.com (D.J.)

**Keywords:** berberine, algae inhibition, hydrogel, sustained-release capsules, response surface methodology

## Abstract

In this study, we used chitosan/sodium alginate hydrogel as a carrier to prepare berberine sustained-release capsule materials that can inhibit algae for a long time and safely. The preparation conditions of the material were optimized by the response surface method, and the optimized capsule material was characterized and the sustained release characteristics were analyzed to study the change of the algae inhibition effect of the material within 30 days. The results showed that the optimum preparation parameters of the material were 0.54% chitosan content, 2.46% sodium alginate content and 1.09% anhydrous calcium chloride content by response surface optimization design, which was consistent with the parameters set by each factor at the central point. The algae inhibition rate of the material under this preparation condition was 93.75 ± 1.01%, which was similar to the predicted value. The release characteristics analysis showed that the material continuously released up to 90% of berberine within 24 days, and its release characteristics were sustained release after burst release, with good sustained release effect. The results of material characterization showed that chitosan/sodium alginate hydrogel could effectively load berberine and was beneficial to the loading and release of berberine. The results of algae inhibition experiments showed that low concentration materials could control the outbreak of cyanobacterial blooms in a short time, while under high concentration conditions, the materials could inhibit *Microcystis aeruginosa* efficiently and for a long time.

## 1. Introduction

With the development of industry and agriculture, a large number of substances containing nitrogen, phosphorus, and other nutrients enter the water body through various ways, resulting in eutrophication of the water body [1]. Some cyanobacteria abnormally reproduce, forming cyanobacteria blooms, destroying the balance of the water ecological environment, and aggravating water pollution, which poses a threat to human health [2,3]. Cyanobacteria bloom has become a global water environmental pollution problem, and its treatment is still a current research hotspot. At present, the technical methods for the treatment of cyanobacterial blooms at home and abroad mainly include physical methods, chemical methods and biological methods [4]. However, the existing research methods often have certain limitations in the practical application process due to their shortcomings. Compared with physical and chemical treatment methods, the biological treatment method of cyanobacterial blooms has lower cost, longer duration and better ecological safety. It has a good application prospect in the treatment of cyanobacterial blooms. The use of allelochemicals to inhibit algae is a new type of biological algae inhibition technology. This technology has low cost, wide material source, high efficiency of algae inhibition and less possibility of secondary pollution [5,6].

Berberine is an alkaloid extracted from *Rhizoma Coptidis*, which belongs to the protoberberine of isoquinoline alkaloids [7]. Studies have shown that berberine has a strong inhibitory effect on *Microcystis aeruginosa*, mainly by inhibiting the photosynthesis of algal cells, damaging its ultrastructure [8] and causing damage to the antioxidant system. Bi et al. [9] used RNA-sequencing technology to study the changes of photosynthesis gene expression of *Microcystis aeruginosa* induced by berberine. It was found that berberine inhibited the photosynthesis of algae by inhibiting the expression of some key genes of photosynthesis. Liu et al. [10] studied the inhibitory effect of berberine on *Microcystis aeruginosa* and its mechanism. The results showed that berberine is a kind of photosynthesis-inhibiting allelochemical, which can selectively inhibit and kill *Microcystis aeruginosa*. The action site is located on the thylakoid photosystem II (PSII), which stimulates the production of a large amount of ROS by hindering the photosynthetic electron transport process of PSII and thus plays a role in inhibiting algae. Zhang et al. [11] found that berberine could induce the antioxidant reaction of *Microcystis aeruginosa* cells, reduce the activity of antioxidant enzymes and lead to oxidative damage of algal cells. However, berberine is hydrophobic and has low solubility in water. The bioavailability of direct addition of berberine is extremely low [12], and large-scale addition of allelochemicals may adversely affect the original environment of the water body and the aquatic ecosystem.

Chitosan and sodium alginate are two kinds of natural polysaccharides. The synthesized chitosan/sodium alginate hydrogel has a certain pore size and a rich network structure [13]. The presence of hydrophilic groups such as amino (-NH_2_), hydroxyl (-OH) and carboxylic acid (-COOH) in the hydrogel can make it have excellent water absorption performance and have a wide range of applications in many fields. In the environmental field, chitosan/sodium alginate hydrogels are often used to remove heavy metal elements and other elements such as nitrogen and phosphorus in water [14,15,16,17,18]. Zhang et al. [19] prepared a chitosan/sodium alginate hydrogel by crosslinking chitosan with sodium alginate and adding Ca^2+^. The material was characterized and analyzed, including scanning electron microscopy, texture analysis and rheological measurement. The obtained material was used for the absorption of dyes in wastewater. The results show that the chitosan/sodium alginate hydrogel has a rich network structure, and the addition of Ca^2+^ promotes the formation of a ‘calcium bridge’ and enhances the properties of the hydrogel, so that it exhibits excellent mechanical strength, strong thermal stability and high adsorption capacity, which can effectively adsorb methylene blue and Congo red in dye wastewater. In addition, chitosan/sodium alginate hydrogels can also be used as drug release carriers to store drugs and control the release rate of drugs [20,21,22,23]. Natalia et al. [24] prepared a highly porous two-component chitosan/sodium alginate aerogel by sol-gel and supercritical fluid technology, which was used as a controlled release drug delivery system. The results show that the obtained material has a developed mesoporous structure, a large specific surface area and high water absorption performance, which is conducive to better loading drugs and prolonging the continuous release time of drugs.

In this study, we used chitosan/sodium alginate hydrogel to load and release berberine to prepare a berberine sustained-release capsule algae inhibitor to improve the bioavailability of berberine and prolong its action time. The effects of main independent variables (chitosan content, sodium alginate content and anhydrous calcium chloride content) on the dependent variable (algae inhibition rate) were analyzed by the response surface method, and the preparation conditions of berberine sustained-release capsules were optimized. In addition, the optimized materials were characterized and the sustained release characteristics were analyzed. Taking *Microcystis aeruginosa* as the treatment target, the algae inhibition effect of the sustained-release capsule material was studied in order to achieve the purpose of safe and long-term algae inhibition, provide a new idea for the treatment of cyanobacteria bloom and provide a theoretical and data reference for the development of new green and lasting allelopathic algae inhibition materials.

## 2. Results and Discussion

### 2.1. Response Surface Optimization Experimental Design and Results

#### 2.1.1. Response Surface Optimization Experimental Data

According to the design concept of the Box–Behnken center combination experiment, the design scheme of the response surface experiment was obtained, and the experimental results were obtained according to the design scheme, as shown in Table 1. In the experimental design scheme, the first, second, eighth, tenth and thirteenth groups are five repeated experiments under central conditions. The purpose is to evaluate the experimental error, and the other groups are factorial experiments to evaluate the effects of three independent variable factors X_1_, X_2_ and X_3_ on the target variable. The experimental results show that the response values of the 17 combined reactions are between 80.34% and 94.72%. Under the conditions of the central experiment, the inhibition rate of berberine sustained-release capsules on *Microcystis aeruginosa* can reach more than 90%. The above table data were analyzed by Design Expert 13 software, and a multiple regression model was established. The inhibition rate of the sustained-release microsphere material was used as the response value Y, and the quadratic polynomial regression equations for X_1_, X_2_ and X_3_ codes were obtained as follows.

(1) $$Y=93.48+0.49*X_{1}-1.00*X_{2}+1.39*X_{3}-0.50*X_{1}*X_{2}+0.11*X_{1}*X_{3}+0.19*X_{2}*X_{3}-1.67*{X_{1}}^{2}-6.41*{X_{2}}^{2}-3.80*{X_{3}}^{2}$$

Final Equation in Terms of Coded Factors:

(2) $$Y=-89.95+37.80*X_{1}+127.33*X_{2}+30.89*X_{3}-4.00*X_{1}*X_{2}+0.88*X_{1}*X_{3}+0.76*X_{2}*X_{3}-26.72*{X_{1}}^{2}-25.62*{X_{2}}^{2}-15.22*{X_{3}}^{2}$$

where Y represents the algae inhibition rate (%) of the sustained-release capsule material. X_1_ is the content of chitosan (*w*/*v*, %). X_2_ is the content of sodium alginate (*w*/*v*, %). X_3_ is the content of anhydrous calcium chloride (*w*/*v*, %).

#### 2.1.2. Model Establishment and Significance Test

The analysis of variance of the regression model in Table 2 shows that the F = 26.35, *p* < 0.0001 of the model, which indicates that the difference of the model is significant. The lack of fit term *p* = 0.2811, which indicates that the lack of fit test of the model is not significant, the model fits well with the actual situation and the model is effective. In addition, the R^2^ value of the model is 0.9713, and the adjusted R^2^ value is 0.9345. The difference between the two is less than 0.2, which indicates that the reliability of the model is high. The coefficient of variation CV = 1.26% < 10%, precision Adeq Precision = 15.012 > 4, which indicates that the model has high accuracy. In this model, X_2_, X_3_, X_1_^2^, X_2_^2^ and X_3_^2^ are all significant items (*p* < 0.05) among all the parameters composed of independent variables X_1_, X_2_ and X_3_; that is, X_2_, X_3_, X_1_^2^, X_2_^2^ and X_3_^2^ are all important model items, which indicates that in the preparation process of berberine sustained-release microsphere materials, the three factors of chitosan content, sodium alginate content and anhydrous calcium chloride content will have a direct impact on the algae inhibition rate of the materials. The results of the F test showed that the degree of influence of these three factors on the inhibitory effect of berberine sustained-release microsphere materials on *Microcystis aeruginosa* was as follows: anhydrous calcium chloride content (X_3_) > sodium alginate content (X_2_) > chitosan concentration (X_1_). In summary, the quadratic regression equation model is more significant and is suitable for analyzing and predicting the optimal preparation conditions of berberine sustained-release microsphere materials.

#### 2.1.3. Response Surface Interaction and Result Analysis

We used Design Expert software to draw the two-dimensional contour map and three-dimensional response surface three-dimensional map of chitosan concentration (X_1_), sodium alginate concentration (X_2_) and calcium chloride concentration (X_3_) on the algae inhibition rate of berberine sustained-release microsphere material. The established quadratic regression equation model was subjected to response surface interaction and result analysis. The surface slope of the response surface stereogram and the offset degree of the contour line can not only reflect the influence of various factors on the response value, but also indicate the interaction between various factors. The tightness of the contour line can also clarify the influence of various factors on the response value [25,26].

**Figure 1:**
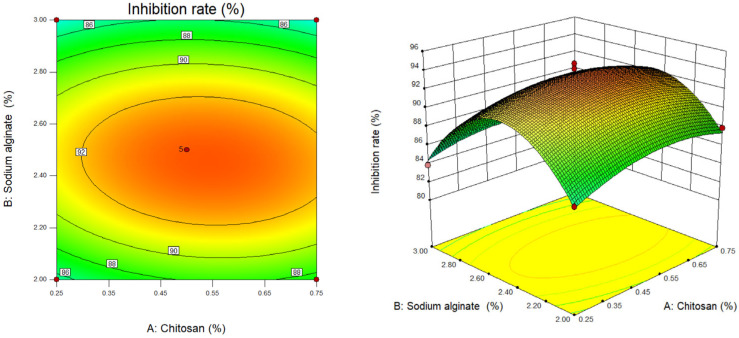
Contour map and response surface map of chitosan content and sodium alginate content.

As shown in Figure 1, when the anhydrous calcium chloride content (X_3_) is at the central point (calcium chloride content is 1%), the response surface slope of chitosan content (X_1_) and sodium alginate content (X_2_) is steep. Combined with the results of the variance analysis of the regression model, X_2_ was significant (*p* > 0.05), indicating that chitosan content (X_1_) and its interaction with sodium alginate content (X_2_) (X_1_X_2_) had little effect on the algae inhibition rate of berberine sustained-release microsphere materials. With the increase of chitosan content (X_1_) and sodium alginate content (X_2_), the algae inhibition rate of sustained-release microsphere materials increased first and then decreased, which indicated that too high or too low chitosan and sodium alginate content were not conducive to the increase of the algae inhibition rate of berberine sustained-release microsphere materials. In addition, it can be seen from the contour map that the contour lines gathered along the direction of the increase of sodium alginate content (X_2_) are denser than those gathered along the direction of the increase of chitosan content (X_1_), which indicates that during the preparation of berberine sustained-release microsphere materials, the effect of sodium alginate content (X_2_) on the algae inhibition rate of microsphere materials is greater than that of chitosan content (X_1_) on the algae inhibition rate of microsphere materials.

**Figure 2:**
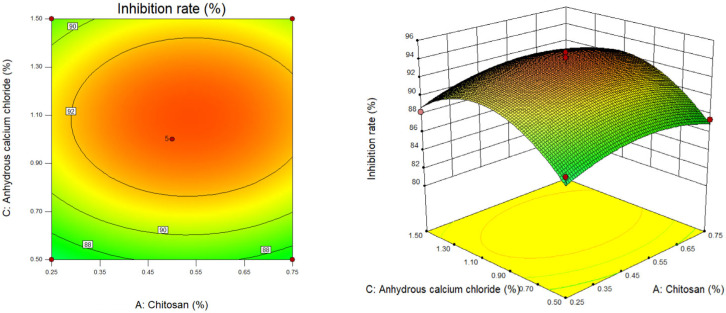
Contour map and response surface map of chitosan content and anhydrous calcium chloride content.

As shown in Figure 2, when the sodium alginate content (X_2_) is at the center point (sodium alginate content is 2.5%), the slope of the response surface of chitosan content (X_1_) and anhydrous calcium chloride content (X_3_) is flat. The results of the regression model analysis of variance showed that X_1_ and X_1_X_3_ were not significant (*p* > 0.05), but X_3_ was significant (*p* < 0.05), which indicated that in the preparation process of sustained-release materials, the effect of anhydrous calcium chloride content (X_3_) on the inhibition rate of berberine sustained-release capsules was greater than that of chitosan content (X_1_) and the interaction between the two (X_1_X_3_). With the increase of chitosan content (X_1_) and anhydrous calcium chloride content (X_3_), the inhibition rate of berberine sustained-release microsphere materials on *Microcystis aeruginosa* showed a trend of increasing first and then decreasing; that is, in the preparation process of sustained-release microsphere materials, too high or too low chitosan content (X_1_) and anhydrous calcium chloride content (X_3_) will lead to a decrease in its inhibition rate. Moreover, it can be seen from the contour map that with the increase of chitosan content (X_1_) and anhydrous calcium chloride content (X_3_), the density of the former contour line is significantly smaller than that of the latter, which indicates that the anhydrous calcium chloride content (X_3_) has a greater impact on the preparation of berberine sustained-release microsphere materials.

**Figure 3:**
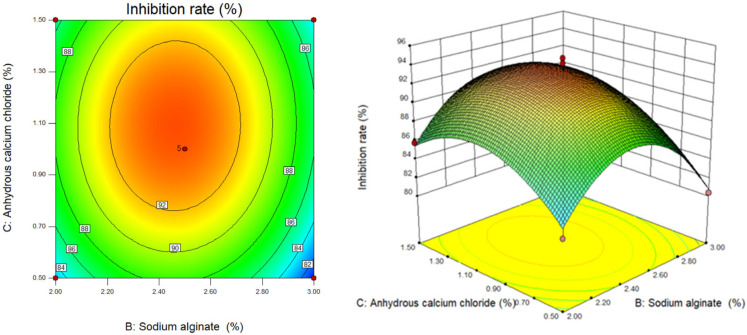
Contour map and response surface map of sodium alginate content and anhydrous calcium chloride content.

As shown in Figure 3, when the chitosan content (X_1_) is at the center point (chitosan content is 0.5%), the response surface slope of sodium alginate content (X_2_) and anhydrous calcium chloride content (X_3_) is very steep. Combined with the analysis of the variance of the regression model, it can be seen that X_2_ and X_3_ are more significant (*p* < 0.05); that is, sodium alginate content (X_2_) and anhydrous calcium chloride content (X_3_) have a significant effect on the algae inhibition rate of berberine sustained-release microsphere materials, and greater than the interaction effect of the two (X_2_X_3_). In the process of material preparation, when the content of sodium alginate (X_2_) and anhydrous calcium chloride (X_3_) increased, the trend of algae inhibition rate of sustained-release microsphere materials increased first and then decreased. In addition, it can be seen from the contour map that the density of the contour line along the direction of the increase of anhydrous calcium chloride content (X_3_) is greater than the density of the contour line along the direction of the increase of sodium alginate content (X_2_), indicating that in the preparation process of sustained-release capsule materials, the influence of anhydrous calcium chloride content (X_3_) on its algae inhibition rate is greater than that of sodium alginate content (X_2_).

In summary, through the regression model analysis of variance and response surface profile analysis, it can be seen that the content of anhydrous calcium chloride has the greatest influence on the algae inhibition rate of berberine sustained-release microsphere materials, while the interaction between sodium alginate content and anhydrous calcium chloride content has little effect on the algae inhibition rate of materials. Combined with the spacing analysis of the contour map, it is concluded that the three influencing factors selected have different significant effects on the algae inhibition rate of berberine sustained-release microsphere materials. The degree of influence is anhydrous calcium chloride content > sodium alginate content > chitosan content.

#### 2.1.4. Determination of Optimum Preparation Conditions and Verification of Optimization Results

**Figure 4:**
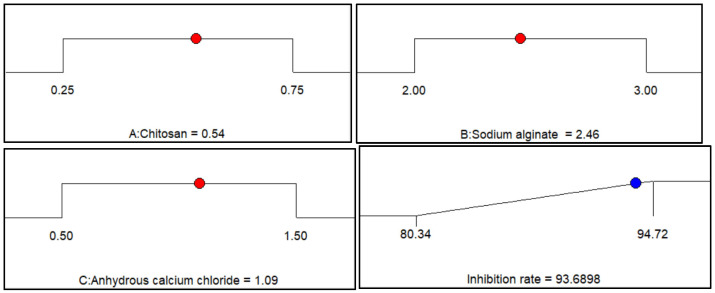
Optimal parameters and results after response surface optimization.

As shown in Figure 4, Design Expert software was used to optimize the preparation conditions of berberine sustained-release microsphere materials. The target response value was set to the maximum value, and then linear fitting and response surface analysis were performed. The optimal preparation parameters were chitosan content 0.54%, sodium alginate content 2.46%, anhydrous calcium chloride content 1.09%. The optimization results are consistent with the parameters set by each influencing factor at the central point. The predicted algae inhibition rate of the berberine sustained-release microsphere material prepared under the optimal conditions is 93.69%.

In order to test the accuracy of the predicted value, three repeated experiments were carried out with the optimized parameters of chitosan content, sodium alginate content and anhydrous calcium chloride content. The algae inhibition rate obtained in the experiment was 93.75 ± 1.01%, which was similar to the predicted value, indicating that the model was feasible, and the model could also predict the algae inhibition rate of berberine sustained-release capsule material on *Microcystis aeruginosa* cells.

### 2.2. Release Curve of Berberine Sustained-Release Microsphere Material

The regression equation of the berberine standard curve was y = 0.0582x + 0.0025 (R^2^ = 0.9998). The results showed that berberine had a good linear relationship in the concentration range of 0.5–25 mg/L, which indicated that ultraviolet spectrophotometry was an effective method for the determination of berberine content.

**Figure 5:**
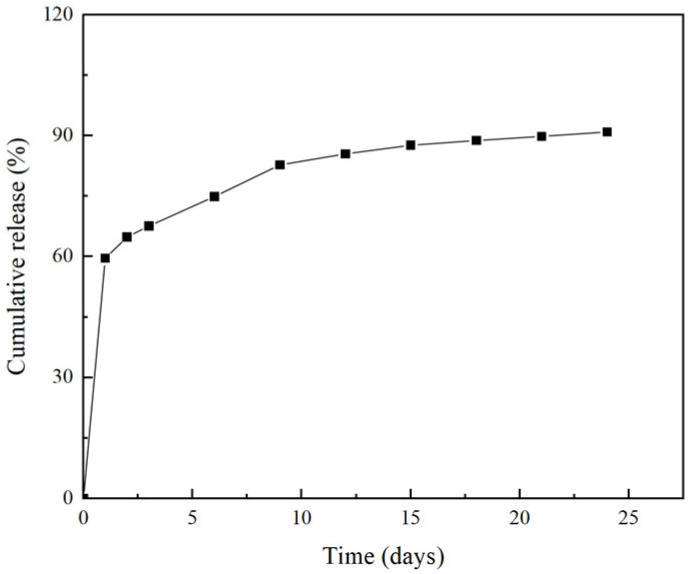
The release curve of berberine sustained-release capsules.

Sustained-release capsules are drug carriers based on natural or synthetic polymer materials, which have the advantages of slow drug release, prolonged drug action time and improved drug stability [27,28,29]. In the process of practical application, in order to ensure the safety and effectiveness of the sustained-release microsphere material, it is necessary to study its release capacity, to obtain a more reasonable delivery scheme and improve the utilization rate of resources. We measured the release of the optimized berberine sustained-release microsphere material within 24 days, and the release curve is shown in Figure 5.

It can be seen from Figure 5 that the content of berberine gradually increased within 24 days and continued to release up to 90% within 24 days, but the level of increase in each time period was different. In the first 24 h of dosing, the content of berberine increased sharply, and the release of berberine reached about 60% after 24 h. With the extension of time, the release of berberine gradually slowed down. This indicates that the release characteristics of berberine sustained-release capsule materials are sustained release after burst release, and have a good sustained release effect [30].

### 2.3. Material Characterization and Analysis

#### 2.3.1. The results of scanning Electron Microscope

**Figure 6:**
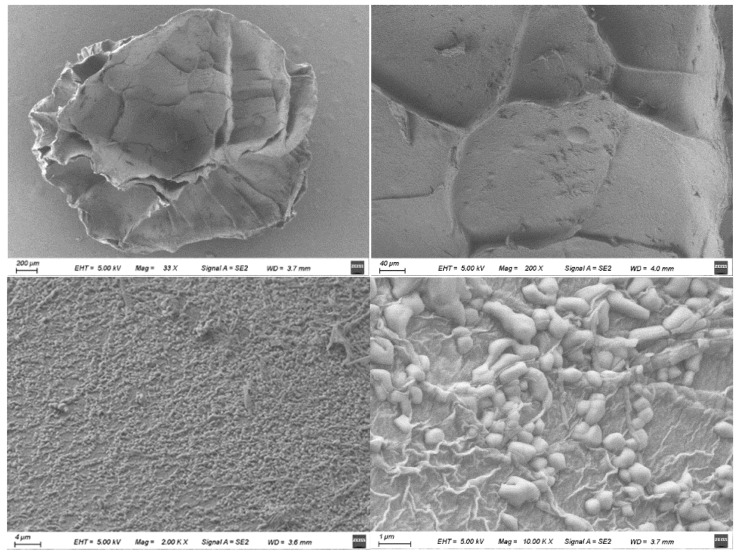
Scanning electron microscopy of berberine sustained-release capsules surface.

**Figure 7:**
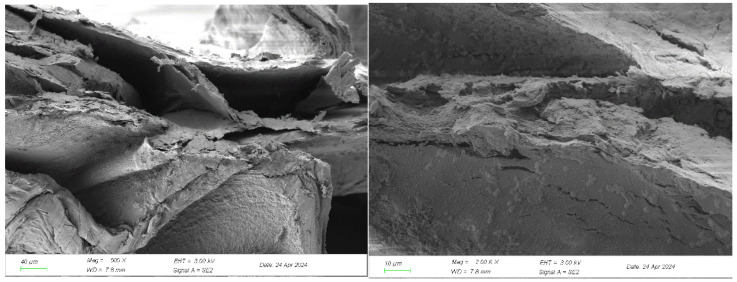
Scanning electron microscopy of the cross-section of berberine sustained-release capsules.

The SEM images of the surface and cross-section of the berberine sustained-release capsule material are shown in Figure 6 and Figure 7.

It can be seen from the surface scanning electron microscope that the berberine sustained-release capsules are oval and have good roundness. The surface of the freeze-dried sustained-release capsules has more wrinkles, less surface damage, good integrity and good loading effect and structural strength. At the same time, it can be seen from the scanning electron microscopy of the cross-section that the material has an interconnected pore structure, which provides a large specific surface area and a large number of active sites, which is conducive to the loading and release of berberine. After magnification, it can be seen that more small particles are agglomerated on its surface. Some studies [31] have found that, in the process of dripping granulation, the uneven rotation speed of magnetic stirring or too small rotation speed will lead to agglomeration.

#### 2.3.2. The results of fourier Transform Infrared Spectroscopy (FT-IR)

**Figure 8:**
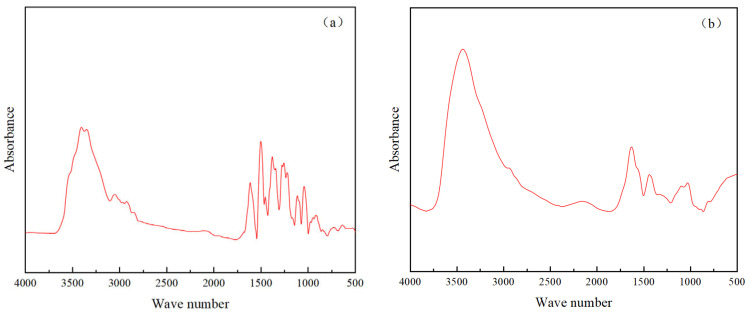
Infrared spectra of berberine (**a**) and berberine sustained-release capsules (**b**).

The infrared spectra of berberine and berberine sustained-release capsule materials are shown in Figure 8a and Figure 8b, respectively.

Berberine is an alkaloid compound with a typical benzene ring structure. It can be seen from the infrared spectrum of berberine (a) that its characteristic peaks are mainly located at 3350, 1614, 1502, 1379, 1257, 1114, 1042 and 724 cm^−1^. Among them, 1614, 1502, 1379, 1257, 1114 and 1042 cm^−1^ contain the skeleton contraction vibration peaks of the benzene ring, 724 cm^−1^ corresponds to the bending vibration of the C-H bond in aromatic compounds and 3350 cm^−1^ is the stretching vibration of -OH and N-H [32]. Chitosan and sodium alginate are natural polymer compounds. Chitosan contains groups such as -NH_2_ and -OH, and sodium alginate contains groups such as -COOH and -OH. In the infrared spectrum of berberine sustained-release capsules (b), the characteristic peaks are mainly located at 3439, 1635, 1439, 1096, 1030 and 805 cm^−1^. The characteristic absorption peak at 3439 cm^−1^ is related to the stretching vibration of -OH and N-H. The characteristic absorption peak at 1635 cm^−1^ is the stretching vibration of -C=O. The characteristic absorption peak at 1439 cm^−1^ is mainly the bending vibration of -CH_3_. The peaks at 1096 and 1030 cm^−1^ belong to the stretching vibration of C-O, and the characteristic absorption peak at 805 cm^−1^ also indicates the existence of a benzene ring. The similar characteristic absorption spectra of the two indicated that the chitosan/sodium alginate hydrogel effectively loaded berberine [33], and there was no chemical interaction between berberine, chitosan and sodium alginate.

### 2.4. The Anti-Algae Effect of Berberine Sustained-Release Microsphere Materials

**Figure 9:**
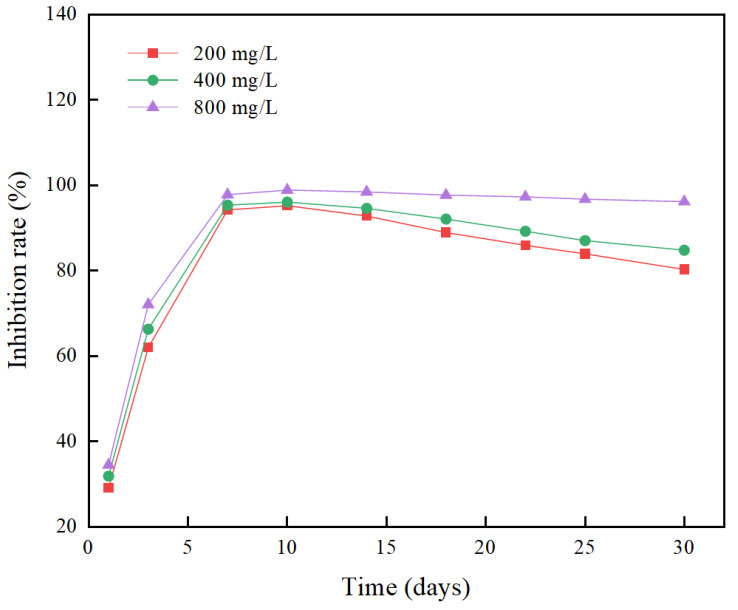
Long-term effects of berberine sustained-release capsules on the growth inhibition rate (IR) of *Microcystis aeruginosa* cells.

Under the long-term algae inhibition experiment of 30 days, the changes in the growth inhibition rate (IR) of different concentrations of berberine sustained-release microsphere materials on *Microcystis aeruginosa* cells are shown in Figure 9. The results showed that each concentration group could effectively inhibit the growth of *Microcystis aeruginosa* cells. In the first 10 days of the experiment, the IR values of each group continued to increase, and the lowest concentration group (200 mg/L) had the highest IR of 95.26%. After 10 days, the inhibitory effect of the low concentration group (200, 400 mg/L) on *Microcystis aeruginosa* cells gradually weakened, and its growth inhibition rate showed a downward trend, but the decline was relatively slow. The IR values were 84.83% and 80.29% on the 30th day, respectively. The IR value of the high-concentration group (800 mg/L) was always at a high level, up to 98.91%, and remained above 90% after 30 days. This result is similar to the conclusion of Lei Zhangyue [34].

In summary, berberine sustained-release capsules have a strong inhibitory effect on *Microcystis aeruginosa*. From the perspective of economic cost-effectiveness, 200 mg/L berberine sustained-release capsules can achieve a good control effect on *Microcystis aeruginosa* in the short term and inhibit the outbreak of cyanobacterial blooms. The 800 mg/L berberine sustained-release capsules had a strong and long-term inhibitory effect on *Microcystis aeruginosa* cells. The berberine sustained-release microsphere algae inhibitor prepared in this paper can indeed inhibit the growth of *Microcystis aeruginosa* for a long time.

## 3. Conclusions

In this study, berberine, an allelochemical with high algae inhibition, was loaded on chitosan/sodium alginate hydrogel by the piercing method [35]. Berberine sustained-release capsules were designed and prepared for long-term and safe algae inhibition. Firstly, the optimal preparation parameters of berberine sustained-release capsule materials optimized by the response surface method were as follows: chitosan content of 0.54%, sodium alginate content of 2.46% and anhydrous calcium chloride content of 1.09%. Secondly, we measured the release of the optimized berberine sustained-release capsule material within 24 days. In general, the release characteristics of the berberine sustained-release capsule material were sustained release after burst release, and it had a good sustained-release effect. The sustained release was as high as 90% within 24 days. Then, we characterized the optimized materials and Fourier transform infrared spectroscopy showed that chitosan/sodium alginate hydrogel could effectively load berberine. Finally, through the algae inhibition experiment, we found that the berberine sustained-release capsule inhibitor had a strong inhibitory effect on *Microcystis aeruginosa*. The 200 mg/L berberine sustained-release capsule material could achieve a good control effect on *Microcystis aeruginosa* in the short term and inhibit the outbreak of cyanobacterial bloom. The 800 mg/L berberine sustained-release capsule algae inhibitor has a strong and long-term inhibitory effect on *Microcystis aeruginosa* cells, which plays an important role in the treatment of cyanobacterial blooms with highly effective algae-inhibiting allelochemicals.

## 4. Materials and Methods

### 4.1. Materials

Berberine was purchased from Shanghai Yien Chemical Technology Co., Ltd. (Shanghai, China), the product number is R014308, the purity is 97%; sodium alginate is chemically pure, purchased from Sinopharm Chemical Reagent Co., Ltd. (Shanghai, China), product number is 30164428; the product number is 9012-76-4, and the degree of deacetylation (DD) is 85.0%. Other reagents used in the experiment, such as anhydrous calcium chloride, acetone, hydrochloric acid, sodium hydroxide, etc., are analytically pure, purchased from Chengdu Cologne Chemical Co., Ltd. (Chengdu, China). The *Microcystis aeruginosa* FACHB912 used in the experiment was purchased from Wuhan Institute of Aquatic Biology, Chinese Academy of Sciences (Wuhan, China). Before the experiment, BG11 medium (purchased from Qingdao Hi-tech Industrial Park Haibo Biotechnology Co., Ltd. (Qingdao, China), product number HB8793) was used to expand the culture reserve.

### 4.2. Preparation of Berberine Sustained-Release Capsules

In this experiment, we used the piercing method to prepare berberine sustained-release microsphere materials. The specific operations are as follows:

(1) A certain amount of sodium alginate and an appropriate amount of ultrapure water were mixed, stirred and ultrasonically dispersed, until the sodium alginate was completely dissolved and cooled.
(2) A certain amount of berberine was mixed with an appropriate amount of ultrapure water, stirred and dispersed by ultrasonic heating until the berberine was completely dissolved and cooled as a core solution for later use.
(3) The sodium alginate solution was adjusted to slightly acidic (pH 4.0) with 1% dilute hydrochloric acid, and mixed with the core solution in a certain volume ratio. After stirring evenly, it was allowed to stand for use; that is, a mixture of core solution and wall solution.
(4) We weighed a certain amount of chitosan and anhydrous calcium chloride, dissolved in 1% (*w*/*v*) glacial acetic acid solution to prepare a fixed solution, and then used 1 mol/L NaOH solution to adjust the pH value of the fixed solution to about 5.5.
(5) The beaker with a fixed solution was placed on a magnetic stirrer and stirred at a low speed. The mixture of the core solution and the wall solution was slowly and uniformly dropped into the fixed solution to form capsules.
(6) After granulation, the capsules were repeatedly washed with ultrapure water, and the superfluous ultrapure water on the surface of the capsules was removed with absorbent paper. The capsules were placed in a freeze-dryer and dried for 48 h.

### 4.3. Design of Response Surface Optimization Experimental

In this study, we selected chitosan content (X_1_), sodium alginate content (X_2_) and anhydrous calcium chloride content (X_3_), which have a great influence on the algae inhibition rate of berberine sustained-release capsule materials, as independent variables, and the algae inhibition rate Y was used as the response value. The Box–Behnken central composite test in the Design Expert software was used to design a three-factor and three-level response surface optimization experiment to further optimize the parameters in the preparation process of berberine sustained-release capsule materials. The factors and level design of the response surface experiment are shown in Table 3.

### 4.4. Determination Method of Berberine Content

The establishment of berberine standard curve [36]: 25 mg berberine was weighed, added with PBS buffer solution to 100 mL, and completely dissolved by ultrasound to obtain a stock solution of 250 mg/L; 0, 0.5, 1, 2, 3, 4, 5 and 10 mL were put into a 50 mL volumetric flask, added with PBS buffer to constant volume and shaken well. Solutions with concentrations of 0, 2.5, 5, 10, 15, 20, 25 and 50 mg/L were obtained, and PBS buffer was used as a blank control. The absorbance was measured at 350 nm.

Determination of samples: In the ultra-clean bench, the sample was shaken, and the appropriate amount was taken in the centrifuge tube. The PBS buffer was used as the blank control, and the absorbance was measured at 350 nm.

### 4.5. Material Characterization

#### 4.5.1. Scanning Electron Microscope

Scanning electron microscope (SEM) uses secondary electrons and backscattered electron signals to obtain information on various physical and chemical properties of the sample itself through vacuum systems, electron beam systems and imaging systems. The equipment model used in this scanning electron microscope is Zeiss GeminiSEM360+ Oxford Energy Spectrum, which is from Carl Zeiss Optics (China) Co., Ltd. (Guangzhou, China), and its resolution is 0.7 nm @ 15 kV (in the case of non-sample stage deceleration mode, no magnetic flux leakage mode); 1.2 nm @ 1 kV (non-sample stage deceleration mode, no magnetic flux leakage mode). Before the identification of the scanning electron microscope, the microsphere material to be tested needs to be dried, and then it is carefully transferred to the conductive adhesive fixed on the surface of the sample table. The nonadherent microsphere material is blown away with the ear-washing ball. The surface of the microsphere material is observed by scanning electron microscopy, and the magnification is adjusted to 8–200 W to obtain a high-resolution image and photograph it.

#### 4.5.2. Fourier Transform Infrared Spectroscopy (FT-IR)

Based on Fourier transform infrared spectroscopy, we analyzed the infrared absorption peaks of berberine and berberine sustained-release capsules to explore the composition of their functional groups. The microsphere material to be tested needs to be dried before Fourier transform infrared spectroscopy, and the background scan is performed with a spectrally pure potassium bromide tablet. The potassium bromide and the sample are ground together and pressed. The spectral range is set to 4000–500 cm^−1^ to determine its absorbance.

### 4.6. The Inhibitory Effect of Berberine Sustained-Release Microsphere Materials on Microcystis aeruginosa

The prepared BG11 medium was placed in a high-pressure steam sterilizer for sterilization (121 °C, 30 min), and placed in an ultra-clean bench for cooling. We poured the cooled BG11 medium into a conical flask and added the algae solution of *Microcystis aeruginosa* to 150 mL. The 30, 60, and 120 mg berberine sustained-release microsphere materials were added to the conical flask, respectively. The groups with only medium and algae solution without other substances were set as blank control groups, and three parallel experiments were set in each group. Taking 30 days as the experimental period, the chlorophyll a (Chl-a) content was measured on the 1st, 3rd, 5th, 7th, 10th, 12th, 14th, 16th, 18th, 20th, 22nd, 25th, 28th and 30th days, and the corresponding algae inhibition rate was calculated.

### 4.7. Inhibition Rate

The inhibition rate of berberine sustained-release microsphere material on the growth of *Microcystis aeruginosa* was calculated by the content of chlorophyll a, and the calculation formula was as follows [37]:

(3) $$IR=\left(1-\frac{N_{t}}{N_{0}}\right)\times 100\%$$

where IR is the growth inhibition rate. N_t_ represents the content of Chl-a in the treatment group (mg/L). N_0_ represents the content of Chl-a in the control group (mg/L).

We determined the content of chlorophyll-a by the acetone extraction method [38]. An appropriate amount of algae solution was centrifuged at 5500 r/min for 10 min in a centrifuge. After centrifugation, the supernatant was discarded, added with 90% acetone and extracted at 4 °C for 24 h. After extraction, the centrifuge was centrifuged again at a speed of 5500 r/min for 10 min. The absorbance was measured at 750, 663, 645 and 630 nm with a spectrophotometer using 90% acetone as a reference. The content of chlorophyll-a was calculated by the following formula:

(4) $$C=11.64\left(A_{663}-A_{750}\right)-2.16\left(A_{645}-A_{750}\right)+0.1(A_{630}-A_{750})$$

(5) $$Chl–a=\frac{CV_{1}}{V_{2}}$$

where A_630_, A_645_, A_663_ and A_750_ represent the absorbance value of the extract at the wavelength of 630, 645, 663 and 750 nm, respectively. V_1_ is the volume of algae liquid (mL). V_2_ is the volume (mL) of 90% acetone. Chl-a represents chlorophyll-a content (mg/L).

### 4.8. Data Analysis

All the above experiments were set up as three parallel experiments, and the experimental results were the average of the three experiments. Microsoft Excel 2021 [39] was used to process the data, and Origin 2021 [40] was used to plot the data. Design Expert software 13 [41] was used to design and analyze the response surface experiment.

## Figures and Tables

**Table 1:** The design and results of the response surface experimental.

Serial Number	X_1_ (%)	X_2_ (%)	X_3_ (%)	Inhibiting Rate (%)
1	0.5	2.5	1	93.25
2	0.5	2.5	1	94.72
3	0.25	2.5	0.5	87.11
4	0.75	2.5	1.5	89.12
5	0.75	2.5	0.5	87.48
6	0.5	2	1.5	85.82
7	0.75	2	1	87.94
8	0.5	2.5	1	92.75
9	0.75	3	1	84.24
10	0.5	2.5	1	94.20
11	0.5	2	0.5	82.04
12	0.25	2.5	1.5	88.31
13	0.5	2.5	1	92.48
14	0.25	3	1	83.87
15	0.5	3	0.5	80.34
16	0.25	2	1	85.57
17	0.5	3	1.5	84.88

Note: X_1_, X_2_ and X_3_ are chitosan content (%), sodium alginate content (%) and anhydrous calcium chloride content (%), respectively.

**Table 2:** Regression model analysis of variance.

Source	Sum of Squares	d/f	Mean Square	F-Value	*p*-Value	
Model	293.13	9	32.57	26.35	<0.0001	Significance
X_1_	1.92	1	1.92	1.55	0.2526	
X_2_	8.08	1	8.08	6.54	0.0377	
X_3_	15.57	1	15.57	12.60	0.0094	
X_1_ X_2_	1.00	1	1.00	0.81	0.3983	
X_1_X_3_	0.048	1	0.048	0.039	0.8488	
X_2_X_3_	0.14	1	0.14	0.12	0.7425	
X_1_^2^	11.74	1	11.74	9.50	0.0178	
X_2_^2^	172.73	1	172.73	139.75	<0.0001	
X_3_^2^	60.96	1	60.96	49.32	0.0002	
Residual	8.65	7	1.24			
Lack of Fit	5.01	3	1.67	1.83	0.2811	No significance
Pure Error	3.64	4	0.91			
Cor Total	301.79	16				
R^2^ = 0.9713; Adjusted R^2^ = 0.9345						
CV = 1.26%; Adeq Precision = 15.012						

Note: X_1_, X_2_ and X_3_ are chitosan content (%), sodium alginate content (%) and anhydrous calcium chloride content (%), respectively.

**Table 3:** Response surface experimental factors and level design.

Factor	Code	The Level of Code		
		−1	0	1
Content of chitosan (*w*/*v*, %)	X_1_	0.25	0.5	0.75
Content of sodium alginate (*w*/*v*, %)	X_2_	2	2.5	3
Content of anhydrous calcium chloride (*w*/*v*, %)	X_3_	0.5	1	1.5

## Data Availability

The original contributions presented in the study are included in the article, further inquiries can be directed to the corresponding author.
